# Asymptomatic and Human-to-Human Transmission of SARS-CoV-2 in a 2-Family Cluster, Xuzhou, China

**DOI:** 10.3201/eid2607.200718

**Published:** 2020-07

**Authors:** Chunyang Li, Fang Ji, Liang Wang, Liping Wang, Jungui Hao, Mingjia Dai, Yan Liu, Xiucheng Pan, Juanjuan Fu, Li Li, Guangde Yang, Jianye Yang, Xuebing Yan, Bing Gu

**Affiliations:** Department of Infectious Disease, Affiliated Hospital of Xuzhou Medical University, Xuzhou, China (C. Li, F. Ji, L. Wang, J. Hao, M. Dai, Y. Liu, X. Pan, J. Fu, L. Li, G. Yang, X Yan);; Department of Bioinformatics, School of Medical Informatics, Xuzhou Medical University, Xuzhou (L. Wang);; Jiangsu Key Laboratory of New Drug Research and Clinical Pharmacy, School of Pharmacy, Xuzhou Medical University, Xuzhou (L. Wang);; Medical Technology School of Xuzhou Medical University, Xuzhou Key Laboratory of Laboratory Diagnostics, Xuzhou (B. Gu);; Department of Laboratory Medicine, Affiliated Hospital of Xuzhou Medical University, Xuzhou (B. Gu)

**Keywords:** COVID-19, 2019 novel coronavirus disease, SARS-CoV-2, severe acute respiratory syndrome coronavirus 2, viruses, respiratory infections, zoonoses, familial cluster, nosocomial transmission, close contact, asymptomatic transmission, Xuzhou, China

## Abstract

We report epidemiologic, laboratory, and clinical findings for 7 patients with 2019 novel coronavirus disease in a 2-family cluster. Our study confirms asymptomatic and human-to-human transmission through close contacts in familial and hospital settings. These findings might also serve as a practical reference for clinical diagnosis and medical treatment.

The ongoing outbreak of 2019 novel coronavirus disease (COVID-19) originating from Wuhan, China, has spread rapidly across the world (*1*). Both human-to-human and asymptomatic transmission have been reported (*2*,*3*). Phylogenetic study reveals that severe acute respiratory syndrome (SARS) coronavirus 2 (SARS-CoV-2), the causative agent of COVID-19, is closely related to 2 SARS-CoV–like bat coronaviruses, bat-SL-CoVZC45 and bat-SL-CoVZXC2 (*4*). Although case-fatality rate for COVID-19 is not finalized yet (*5*), it is largely accepted that the infection is less fatal than that for SARS-CoV infection, which had an ≈10% case-fatality rate (*6*). 

Typical symptoms of COVID-19 include fever, cough, and fatigue, whereas sputum, headache, hemoptysis, and diarrhea are less common (*7*). No vaccine to prevent the infection exists. In this study, we describe a cluster of 7 COVID-19 case-patients among whom interfamilial and intrafamilial transmission occurred. Our findings are consistent with previous confirmation of asymptomatic and human-to-human transmission of SARS-CoV-2 in family and hospital settings and also provide practical reference for clinical diagnosis and treatment of COVID-19.

On January 14, 2020, a 56-year-old man (index patient) departed from Guangzhou, China, transferred at Hankou Station in Wuhan, China, for 6 hours, and arrived at Xuzhou, China, showing no symptoms on the same day in the evening. During January 14–22, he had close contact with his 2 daughters, a 32-year-old pregnant teacher (patient 1) and a 21-year-old undergraduate student (patient 2). On January 15, he began caring for his 42-year-old son-in-law (patient 3, husband of patient 1), who had been hospitalized at the Affiliated Hospital of Xuzhou Medical University in Xuzhou until January 23. Meanwhile, a 62-year-old man (patient 4) stayed in the hospital during January 2–19 because of pancreatic surgery; he shared the same ward with patient 3 and was cared for by his 34-year-old son (patient 5). During January 15–January 18, patients 4 and 5 had close contact with the index patient, who was asymptomatic during that time. On January 19, patient 4 was discharged to home and had close contact with his 56-year-old wife (patient 6). We compiled a comprehensive illustration of the contact history of the clustered cases (Figure).

**Figure:**
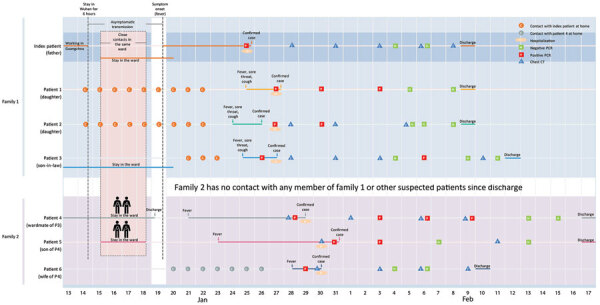
Chronology of a 2-family cluster of severe acute respiratory syndrome coronavirus 2 infection, including travel and contact history, in familial and hospital settings, Xuzhou, China, January 13–February 17, 2020. Dates of case confirmation, hospitalization, and discharge are labeled. Real-time fluorescent reverse transcription PCR for severe acute respiratory syndrome coronavirus 2 infection and corresponding results are indicated, together with the dates of chest CT. CT, computed tomography.

On January 25, the index patient was confirmed to have COVID-19 and was admitted to the Affiliated Hospital of Xuzhou Medical University with symptoms of fever, cough, and sore throat. His illness rapidly became severe; he had a high respiratory rate (38 breaths/min) and low oximetry saturation (<93%). Subsequently, during January 26–31, another 6 members of the 2 families all tested positive for SARS-CoV-2 by real-time fluorescent reverse transcription PCR of their throat swab samples. The clinical features of these patients varied (Appendix Table 1).

We used imaging features of pneumonia (detected using chest computed tomography) as clinical confirmation for all patients except patient 1. We performed laboratory diagnostic tests, including routine blood tests, comprehensive metabolic panels, coagulation tests, and screening for infection for all patients (Appendix Tables 2–4). We provided all patients with medical therapy (Appendix Table 5, Figure 1) except patient 1, who was pregnant. Because the index patient was in severe condition during his hospitalization, we have included a more detailed description of his medical treatment. 

During January 26–February 3, we administered to the index patient the antiviral drugs lopinavir/ritonavir (400 mg/100 mg 2×/d by mouth), umifenovir (200 mg 3×/d by mouth), and interferon α-2b (5 MIU 2×/d by aerosolized inhalation). We administered the antibacterial drug moxifloxacin hydrochloride (400 mg 1×/d by intravenous drip) during January 28–February 6, 2020, and intravenous immunoglobulin therapy (20 g/d) during January 28–February 1. In addition, we administered glucocorticoid therapy with methylprednisolon (20–60 mg 2×/d by intravenous drip) during January 29–February 1. The patient’s fever abated on January 29. He tested negative for SARS-CoV-2 on February 4 and again on February 6. During the progression of his recovery, we observed gradual reduction of the white patches in the lung caused by SARS-CoV-2 infection (Appendix Figure 2). On January 28 and January 31, we observed multiple ground-glass–like high-density shadows on both lungs with blurred edges and interstitial changes. On February 3, high-density shadows were slightly absorbed in the upper lobe of the bilateral lungs. On February 6, some lesions in the lower lobe of both lungs were slightly absorbed, and we observed the same situation on February 8. The index patient was discharged to home on February 9.

In summary, our epidemiologic study demonstrates asymptomatic and human-to-human transmission of SARS-CoV-2 infection through close contacts in both familial and hospital settings. In addition, the laboratory test results, together with course of medical therapies described, can provide a practical reference for COVID-19 diagnosis and treatment.

Appendix Additional information about asymptomatic and human-to-human transmission of SARS-CoV-2 in a 2-family cluster, Xuzhou, China.
