# Supplementary material for: Asymptomatic and Human-to-Human Transmission of SARS-CoV-2 in a 2-Family Cluster, Xuzhou, China
Source: Emerg Infect Dis. 2020 Jul;26(7):1626–8. doi: 10.3201/eid2607.200718 (PMC7323514; doi:10.3201/eid2607.200718)
Supplement: Appendix — Additional information about asymptomatic and human-to-human transmission of SARS-CoV-2 in a 2-family cluster, Xuzhou, China. [file 20-0718-Techapp-s1.pdf]

Article DOI: https://doi.org/10.3201/eid2607.200718

Appendix

Asymptomatic and Human-to-Human Transmission of SARS-CoV-2 in a 2-Family Cluster, Xuzhou, China

Appendix Table 1. Summary of clinical features of all cases in the two-family cluster infected with SARS-CoV-2019 before hospitalization.

		Index Patient	Patient 1	Patient 2	Patient 3	Patient 4	Patient 5	Patient 6
Relationship		Father of P1 and P2	Daughter of Index Patient	Daughter of Index Patient	Son-in-law of Index Patient	Wardmate of P3	Son of P4	Wife of P4
Age (years)		56	32	21	42	62	34	56
Sex		Male	Female	Female	Male	Male	Male	Female
Occupation		Farmer	Teacher	Undergraduate student	Scientific Researcher	Farmer	Worker	Farmer
Date of symptom onset		19/1/2020	25/1/2020	24/1/2020	25/1/2020	21/1/2020	23/1/2020	28/1/2020
Date of hospital admission		25/1/2020	27/1/2020	27/1/2020	27/1/2020	28/1/2020	30/1/2020	30/1/2020
Date of case confirmation		25/1/2020	27/1/2020	26/1/2020	27/1/2020	29/1/2020	31/1/2020	30/1/2020
Interval between symptom onset and case confirmation		7	3	3	3	9	9	3
History of smoking		None	None	None	None	None	None	None
History of alcohol drinking		None	None	None	None	None	None	None
Chronic medical illness		None	Pregnancy	None	None	Hypertension	None	Diabetes, Breast Cancer, Cervical cancer
Presenting symptoms and signs before admitted to hospital	Fever	+	–	+	+	+	+	+
	Peak body temperature (°C)	39.5	37.3	37.5	38	39.2	37.5	37.3
	Cough	–	–	+	–	–	+	–
	Sputum	–	–	+	–	–	+	+
	Shortness of breath	–	–	–	–	+	–	+
	Breath difficulty	–	–	–	–	–	–	+
	Sore throat	+	–	–	–	–	–	–
	Headache	–	–	–	–	–	–	–
	Vomit	+	–	–	–	–	–	+
	Diarrhea	–	–	–	–	–	–	+
	Muscular Soreness	–	–	–	–	–	–	–
	Fatigue	+	–	–	–	–	–	+
	Oximetry saturation (%)	92	99	95	95	99	95	100
	Respiratory rate (breaths/min)	23	15	16	22	20	18	32
	Blood pressure (mmHg)	130/70	115/72	120/80	120/80	147/87	123/76	98/70
	Heart rate (bpm)	76	72	72	75	123	85	60

Appendix Table 2. Summary of medical laboratory tests for index patient in the two-family cluster.

Laboratory Diagnosis		Index Patient				
Routine Blood Test	Normal Range		29/1/20	31/1/20	3/2/20	8/2/20
Leukocytes (10^9/L)	4.0–10.0		4.9	10.3	5.6	7.1
Neutrophil Ratio (%)	50.0–70.0		86	89.5	77.9	77.1
Lymphocyte Ratio (%)	20.0–40.0		10.8	6.4	13.9	13
Monocyte Ratio (%)	3.0–8.0		3.1	4	8.2	9
Neutrophil Count (10^9/L)	2.00–7.00		4.23	9.23	4.39	5.47
Lymphocyte Count (10^9/L)	0.80–4.00		0.5	0.7	0.8	0.9
Monocyte Count (10^9/L)	0.12–1.20		0.15	0.41	0.46	0.64
Red blood cell Count (10^12/L)	3.5–5.0		4.68	4.34	4.62	4.49
Hemoglobin (g/L)	110–150		154	140	148	146
Hematocrit (%)	37.0–43.0		44	40.9	41.7	43.4
Platelet Count (10^9/L)	100–300		178	262	282	248
Red blood cell Distribution Width (%)	11.6–14.0		12.1	12.4	12	12.1
Mean Platelet volume (fL)	9.4–12.5		11.1	11.8	12	10.1
Platelet Distribution Width (fL)	39.0–46.0		16.6	13.7	14.5	16.3
Platelet Hematocrit (%)	0.108–0.282		0.2	0.31	0.34	0.25
Comprehensive Metabolic Panel	Normal Range	26/1/20	28/1/20	31/1/20	3/2/20	8/2/20
Alanine Aminotransferase (U/L)	0–45	23	29	55	27	33
Aspartate Aminotransferase (U/L)	0–40	30	36	30	15	19
Alkaline Phosphatase (U/L)	42–128	80	83	68	61	77
Glutamyltransferase (U/L)	11–50	95	101	89	73	87
Lactate Dehydrogenase (U/L)	0–252	334	403	295	229	195
Total Bilirubin (umol/L)	0–20	9.8	14.5	11	21.6	9.9
Albumin (g/L)	34–48	41.4	41.8	33.5	34	35.6
Glucose (mmol/L)	3.8–6.1	/	/	/	5.83	6.22
Urea (mmol/L)	1.7–8.3	6.1	6.4	7.2	7.1	4.5
Creatinine (umol/L)	44–97	122	112	72	79	76
Uric Acid (umol/L)	208–428	373	439	273	264	259
Triglyceride (mmol/L)	0–1.70	2.25	/	1.66	4.22	4.93
Total Cholesterol (mmol/L)	2.80–5.20	4.19	/	4.06	4.34	4.71
Calcium (mmol/L)	2.1–2.7	2.28	2.24	2.19	2.12	2.17
Phosphorus (mmol/L)	.097–1.61	0.98	1.49	0.84	0.75	/
Potassium (mmol/l)	3.5–5.3	4.61	4.27	4.63	3.89	4.24
Sodium (mmol/l)	135–146	141	135	137.2	134.2	145
Chlorine (mmol/l)	96–108	101.4	100.2	107.1	97.3	104.4
eGFR (ml/min/1.73m^2)	100–120	/	62.53	104.12	93.55	/
Infection Test	Normal Range	23/1/20	28/1/20	31/1/20	3/2/20	8/2/20
Erythrocyte Sedimentation Rate (mm/1h)	M 0–15/F 0–20	/	/	/	/	/
Ferritin (μg/L)	M 0–322/F 0–219	/	1253	929.65	881.9	/
Procalcitonin (ng/ml)	0–0.1	/	0.08	0.04	0.04	0.06
C-reactive Protein (mg/L)	0.8–8	101.4	/	7.9	1.6	6.4
Coagulation	Normal Range	26/1/20	28/1/20	31/1/20		8/2/20
International Normalized Ratio	0.8–1.2	1.08	1.15	1.04		1
Prothrombin Time (s)	10–14	11.7	12.4	11.2		10.8
Activated Partial Prothrombin Time (s)	21–40	36.6	36.1	30.8		28.9
Thrombin Time (s)	14.0–21	12.6	12.8	14.7		14.9
D-dimer (μg/ml)	0–0.5	0.14	0.13	0.37		0.59

Appendix Table 3. Summary of medical laboratory tests for patients 1–3 in the two-family cluster.

Laboratory Diagnosis		Patient 1			Patient 2		Patient 3	
Routine Blood Test	Normal Range	27/1/20	3/2/20		3/2/20	27/1/20	1/2/20	6/2/20
Leukocytes (10^9/L)	4.0–10.0	3.7	3.9		12.6	5.9	2.4	6.9
Neutrophil Ratio (%)	50.0–70.0	72.6	66.1		68.4	63.6	69.3	48.5
Lymphocyte Ratio (%)	20.0–40.0	16.5	24.2		26.1	21.6	28.2	38.7
Monocyte Ratio (%)	3.0–8.0	10.6	8.2		4.8	12.4	2.2	11.5
Neutrophil Count (10^9/L)	2.00–7.00	2.68	2.57		8.59	3.75	1.66	3.36
Lymphocyte Count (10^9/L)	0.80–4.00	0.6	0.9		3.3	1.3	0.7	2.7
Monocyte Count (10^9/L)	0.12–1.20	0.39	0.32		0.6	0.73	0.05	0.79
Red blood cell Count (10^12/L)	3.5–5.0	3.23	3.02		5.06	4.3	4.48	4.13
Hemoglobin (g/L)	110–150	106	98		141	116	123	112
Hematocrit (%)	37.0–43.0	30.2	28		42.2	36.1	37	35.1
Platelet Count (10^9/L)	100–300	142	174		346	647	499	447
Red blood cell Distribution Width (%)	11.6–14.0	11.9	11.6		11.7	13	12.4	12.6
Mean Platelet volume (fL)	9.4–12.5	10.2	10.2		9.3	10	9.2	9.2
Platelet Distribution Width (fL)	39.0–46.0	10.8	10.8		10.1	10.4	15.7	15.7
Platelet Hematocrit (%)	0.108–0.282	0.15	0.16		0.32	0.71	0.46	0.41
Comprehensive Metabolic Panel	Normal Range	27/1/20	3/2/20	27/1/20	3/2/20	27/1/20	1/2/20	6/2/20
Alanine Aminotransferase (U/L)	0–45	14	8	25	11	47	36	28
Aspartate Aminotransferase (U/L)	0–40	18	13	33	12	30	27	15
Alkaline Phosphatase (U/L)	42–128	71	78	70	63	65	76	46
Glutamyltransferase (U/L)	11–50	9	8	40	23	41	39	29
Lactate Dehydrogenase (U/L)	0–252	111	113	220	162	197	228	/
Total Bilirubin (umol/L)	0–20	5.9	3.3	8.8	9.5	6	8.5	7.7
Albumin (g/L)	34–48	35.7	30.9	49.1	43.7	40.1	43.7	30.4
Glucose (mmol/L)	3.8–6.1	/	4.44	/	4.06	/	/	/
Urea (mmol/L)	1.7–8.3	2.8	2.9	3.6	2.4	3.5	3.1	5.1
Creatinine (umol/L)	44–97	42	40	60	56	63	63	56
Uric Acid (umol/L)	208–428	300	252	487	413	271	253	233
Triglyceride (mmol/L)	0–1.70	1.82	2.87	1.17	3.44	0.87	1.08	2.92
Total Cholesterol (mmol/L)	2.80–5.20	5.14	4.61	3.41	3.66	3.63	4.96	4.2
Calcium (mmol/L)	2.1–2.7	2.09	1.97	2.34	2.19	2.22	2.32	2.02
Phosphorus (mmol/L)	.097–1.61	1.32	1.12	1.46	1.05	1.32	1.31	1.24
Potassium (mmol/l)	3.5–5.3	3.58	3.41	3.75	3.76	4.81	4.99	4.44
Sodium (mmol/l)	135–146	136.5	138	139.7	138.8	134.8	133.4	138
Chlorine (mmol/l)	96–108	103.6	105.4	103.9	102.9	98.9	96.7	102.6
eGFR (ml/min/1.73m^2)	100–120	/	>120	/	>120	/	>120	>120
Infection Test	Normal Range	27/1/20	3/2/20	27/1/20	3/2/20	27/1/20	1/2/20	6/2/20
Erythrocyte Sedimentation Rate (mm/1h)	M 0–15/F 0–20	/	47	/	28	/	50	14
Ferritin (µg/L)	M 0–322/F 0–219	/	14.16	/	128.2	/	/	/
Procalcitonin (ng/ml)	0–0.1	0.04	0.06	0.05	0.03	0.06	0.04	/
C-reactive Protein (mg/L)	0.8–8	16.3	/	/	/	4.4	7.1	0.8
Coagulation	Normal Range	27/1/20	3/2/20	27/1/20	3/2/20	27/1/20	1/2/20	6/2/20
International Normalized Ratio	0.8–1.2	0.99	0.94	1.08	1.01	1.23	1.12	1.1
Prothrombin Time (s)	10–14	10.7	10.1	11.7	10.9	13.3	12.1	11.9
Activated Partial Prothrombin Time (s)	21–40	26.7	28.9	27.9	31.7	30.7	30.6	31.6
Thrombin Time (s)	14.0–21	13.1	15.4	13.2	15.1	15.1	14.6	15.3
D-dimer (µg/ml)	0–0.5	0.21	0.28	0.07	0.06	0.51	0.48	0.69

Appendix Table 4. Summary of medical laboratory tests for patients 4–6 in the two-family cluster

Laboratory Diagnosis		Patient 4					Patient 5		Patient 6		
Routine Blood Test	Normal Range	28/1/20	2/2/20	4/2/20	6/2/20	10/2/20	30/1/20	3/2/20	30/1/20	3/2/20	8/2/20
Leukocytes (10^9/L)	4.0–10.0	8.2	15.9	17.7	14.3	5.9	4.5	4.2	2.4	4.2	3.7
Neutrophil Ratio (%)	50.0–70.0	80.5	78.6	87.9	83.6	71.1	54.1	49.9	71.4	59.8	53.5
Lymphocyte Ratio (%)	20.0–40.0	7.9	14.1	7.4	10.8	22.1	36.1	40.8	26.6	32.6	35.5
Monocyte Ratio (%)	3.0–8.0	11.3	6.6	4	4.7	5.2	8.6	7.2	1.7	7.4	7.8
Neutrophil Count (10^9/L)	2.00–7.00	6.57	12.47	15.56	11.96	4.19	2.43	2.09	1.69	2.51	1.98
Lymphocyte Count (10^9/L)	0.80–4.00	0.7	2.2	1.3	1.5	1.3	1.6	1.7	0.6	1.4	0.29
Monocyte Count (10^9/L)	0.12–1.20	0.92	1.05	0.71	0.67	0.31	0.38	0.3	0.04	0.31	0.12
Red blood cell Count (10^12/L)	3.5–5.0	2.26	2.61	3.39	3	2.68	4.64	265	3.97	4.45	4.01
Hemoglobin (g/L)	110–150	75	86	113	102	90	146	150	124	134	125
Hematocrit (%)	37.0–43.0	21.5	25.6	33.2	29.3	25.8	40.9	43	35.6	38.8	36.3
Platelet Count (10^9/L)	100–300	240	365	407	378	313	213	265	136	164	194
Red blood cell Distribution Width (%)	11.6–14.0	11.4	11.9	11.9	12.3	12.7	11.2	11.2	11.9	11.9	11.1
Mean Platelet volume (fL)	9.4–12.5	8.6	8.1	7.8	8.1	7.9	7.9	7.7	8.6	9.9	8.2
Platelet Distribution Width (fL)	39.0–46.0	15.7	15.5	15.5	15.7	15.5	15.7	15.7	16.2	10.9	16
Platelet Hematocrit (%)	0.108–0.282	0.21	0.3	0.32	0.31	0.25	0.17	0.2	0.12	0.16	0.16
Comprehensive Metabolic Panel	Normal Range	28/1/20	2/2/20	4/2/20	6/2/20	10/2/20	30/1/20	3/2/20	30/1/20	3/2/20	8/2/20
Alanine Aminotransferase (U/L)	0–45	28	24	/	27	22	45	33	22	24	24
Aspartate Aminotransferase (U/L)	0–40	22	14	/	19	19	30	27	25	27	22
Alkaline Phosphatase (U/L)	42–128	175	99	/	64	68	56	48	121	111	113
Glutamyltransferase (U/L)	11–50	123	66	/	43	38	23	18	33	28	28
Lactate Dehydrogenase (U/L)	0–252	129	133	/	187	144	126	128	163	166	175
Total Bilirubin (umol/L)	0–20	10.3	8.7	/	10.7	8.3	27.6	14	11.9	11	3.7
Albumin (g/L)	34–48	24.6	31.8	/	41.5	45.2	46.7	44	43.3	45.1	36.1
Glucose (mmol/L)	3.8–6.1	6.73	/	/	/	2.87	6.88	5.01	10.67	5.18	5.87
Urea (mmol/L)	1.7–8.3	4.1	4.5	/	8	7.2	3.7	4.6	4.6	6.3	4.2
Creatinine (umol/L)	44–97	59	55	/	39	53	60	65	51	54	37
Uric Acid (umol/L)	208–428	175	145	/	156	211	309	246	278	321	102
Triglyceride (mmol/L)	0–1.70	0.89	1.43	/	2.87	0.87	/	2.17	/	3.51	6.52
Total Cholesterol (mmol/L)	2.80–5.20	2.25	2.65	/	2.46	2.22	/	3.72	/	5.45	4.47
Calcium (mmol/L)	2.1–2.7	1.86	2.22	2.11	2.24	2.32	2.26	2.19	2.19	2.12	2.18
Phosphorus (mmol/L)	.097–1.61	/	/	1	0.9	1.15	1.06	1.1	1	1.22	/
Potassium (mmol/l)	3.5–5.3	3.35	4.93	5.27	4.56	4.28	4.05	4.47	4.21	3.12	3.54
Sodium (mmol/l)	135–146	131	138	135	132.4	138.9	138.4	139.5	140.2	141.9	148
Chlorine (mmol/l)	96–108	90	99.2	96	97.1	98.1	102.9	104	107	101.2	106.9
eGFR (ml/min/1.73m^2)	100–120	/	/	/	>120	>120	>120	>120	115.02	107.68	/
Infection Test	Normal Range	28/1/20	31/1/20	4/2/20	6/2/20	10/2/20	30/1/20	3/2/20	30/1/20	3/2/20	8/2/20
Erythrocyte Sedimentation Rate (mm/1h)	M 0–15/F 0–20	/	/	/	/	11	8	/	14	/	/
Ferritin (μg/L)	M 0–322/F 0–219	/	564	773.6	892.7	/	248.5	237	339.4	490.6	/
Procalcitonin (ng/ml)	0–0.1	0.6	/	/	0.08	0.1	0.03	0.02	0.04	0.02	0.06
C-reactive Protein (mg/L)	0.8–8	174.6	/	17.6	7.4	4.2	0	0	1.8	17	3.5
Coagulation	Normal Range	28/1/20	2/2/20		6/2/20	10/2/20	30/1/20	3/2/20	30/1/20		8/2/20
International Normalized Ratio	0.8–1.2	1.51	1.16		1.28	1.38	1.21	1.12	1.17		1.03
Prothrombin Time (s)	10–14	16.3	12.5		13.8	14.9	13.1	12.1	12.6		11.1
Activated Partial Prothrombin Time (s)	21–40	26	24.6		26.3	29	30.2	31.7	28		28.3
Thrombin Time (s)	14.0–21	15.8	16.5		15.2	16.4	17.2	19	17		16.8
D-dimer (μg/ml)	0–0.5	0.76	0.84		1.07	0.58	0.01	0.06	0.05		0.09

Appendix Table 5. Summary of drug therapy during COVID-19 treatment of the clustered cases. Due to pregnancy, patient 1 did not receive any medication. For an illustration of the drug therapy scheme, please see the visualized timeline below.

Index Patient	Dosage and Administration	Start Date	End Date
Lopinavir/Ritonavir	400mg/100mg bid po	26/1/20	3/2/20
Umifenovir	200mg tid po	29/1/20	3/2/20
Interferon α-2b	5MIU bid aerosolized inhalation	29/1/20	3/2/20
Moxifloxacin hydrochloride	0.4g qd ivgtt	28/1/20	6/2/20
Immunoglobulin	20 g/d	28/1/20	1/2/20
Methylprednisolon	40mg bid ivgtt	28/1/20	29/1/20
Methylprednisolon	60mg qd ivgtt	30/1/20	31/1/20
Methylprednisolon	40mg qd ivgtt	31/1/20	1/2/20
Methylprednisolon	20mg qd ivgtt	31/1/20	1/2/20
Patient 2	Dosage and Administration	Start Date	End Date
Lopinavir/Ritonavir	400mg/100mg bid po	31/1/20	6/2/20
Umifenovir	200mg tid po	29/1/20	6/2/20
Moxifloxacin hydrochloride	0.4g qd po	27/1/20	2/2/20
Ketotifen fumarate	1mg qd qn	27/1/20	2/2/20
Patient 3	Dosage and Administration	Start Date	End Date
Lopinavir/Ritonavir	400mg/100mg bid po	30/1/20	5/2/20
Umifenovir	200mg tid po	29/1/20	6/2/20
Interferon α-2b	5MIU bid aerosolized inhalation	31/1/20	5/2/20
Moxifloxacin hydrochloride	0.4g qd po	31/1/20	6/2/20
Methylprednisolon	40mg bid ivgtt	1/2/20	3/2/20
Lianhua Qingwen capsule	6g tid	31/1/20	12/2/20
Budesonide	1mg qd aerosolized inhalation	31/1/20	5/2/20
Patient 4	Dosage and Administration	Start Date	End Date
Lopinavir/Ritonavir	400mg/100mg bid po	30/1/20	3/2/20
Umifenovir	200mg tid po	29/1/20	4/2/20
Interferon α-2b	5MIU bid aerosolized inhalation	30/1/20	6/2/20
Biapenem	0.3g q8h ivgtt	29/1/20	6/2/20
Linezolid	0.6g q12h ivgtt	5/2/20	13/2/20
Patient 5	Dosage and Administration	Start Date	End Date
Lopinavir/Ritonavir	400mg/100mg bid po	30/1/20	1/2/20
Umifenovir	200mg tid po	30/1/20	6/2/20
Moxifloxacin hydrochloride	0.4g qd po	30/1/20	6/2/20
Patient 6	Dosage and Administration	Start Date	End Date
Lopinavir/Ritonavir	400mg/100mg bid po	30/1/20	2/2/20
Umifenovir	200mg tid po	30/1/20	6/2/20
Interferon α-2b	5MIU bid aerosolized inhalation	31/1/20	6/2/20
Moxifloxacin hydrochloride	400mg qd po	26/1/20	7/2/20
Methylprednisolon	40mg bid ivgtt	30/1/20	30/1/20
Chinese medicine decoction	1Package bid po	5/2/20	10/2/20
Lianhua Qingwen capsule	6000mg tid po	8/2/20	10/2/20

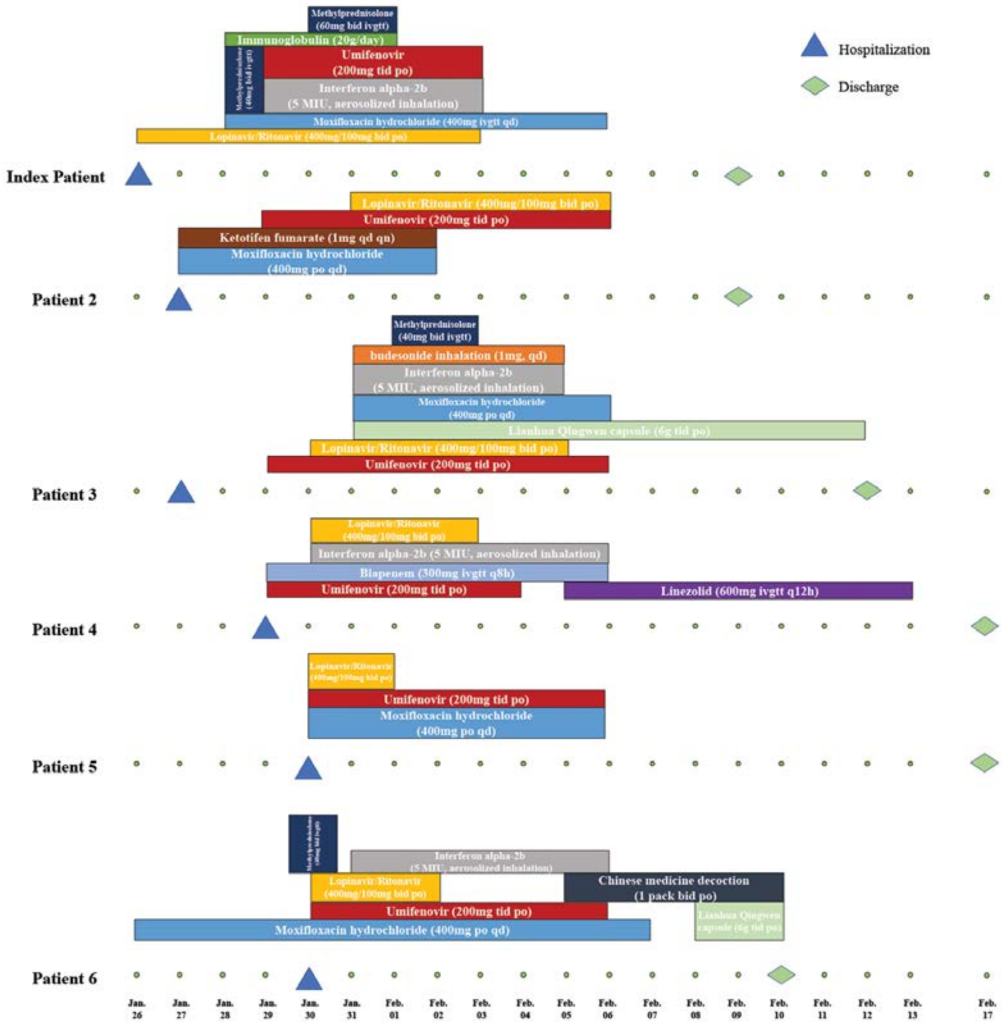

Appendix Figure 1. Illustration of the drug therapy scheme for all the patients with SARS-CoV-2 infection in the 2-family cluster.

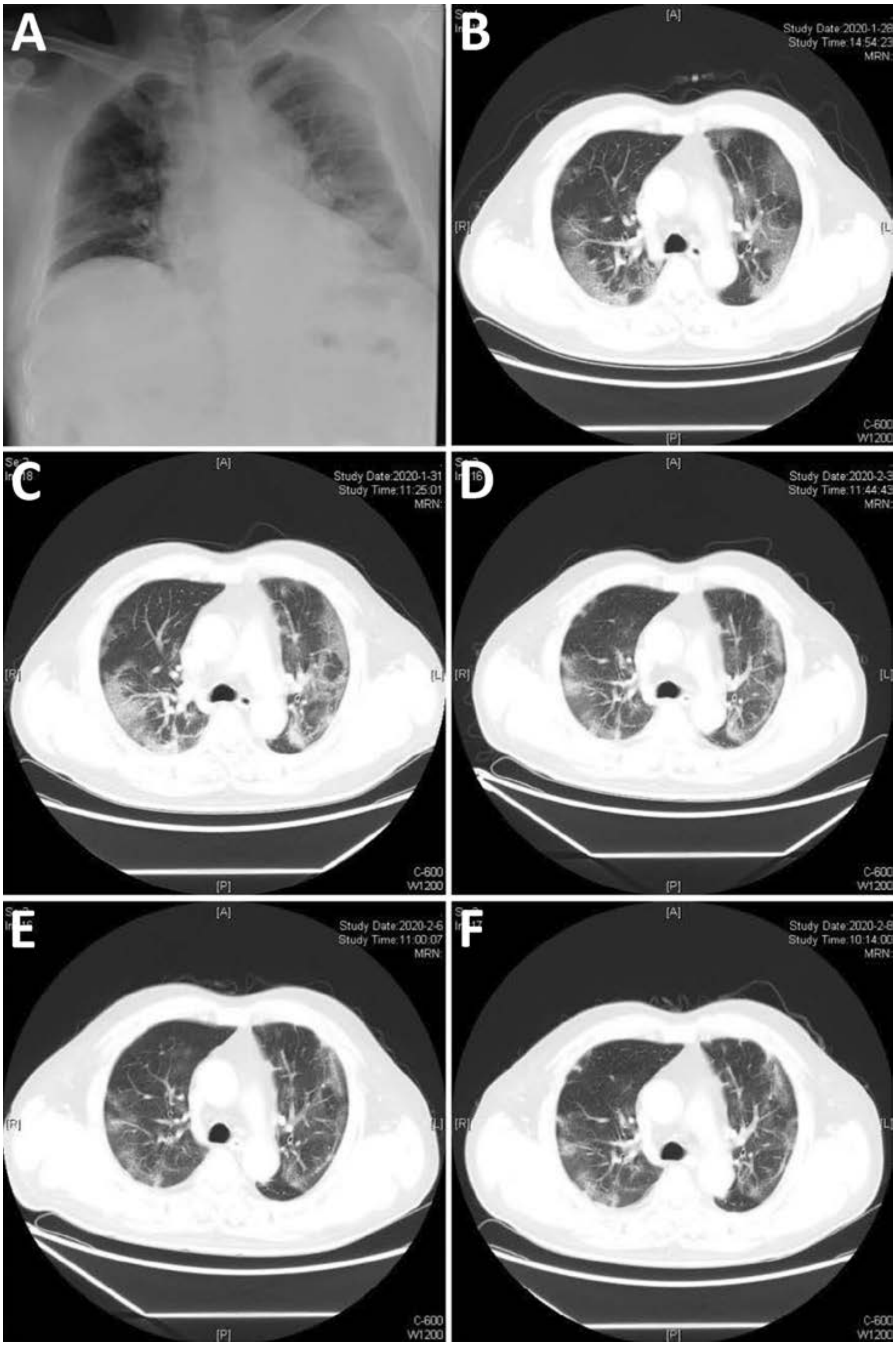

Appendix Figure 2. Typical evolution of chest CT findings in the 56-year-old male index patient confirmed positive for SARS-CoV-2 infection. A) Chest radiograph. Bilateral lungs show flocculent high-density shadows while the left lung is more prominent. B–F) Chest CT scanning. B) Multiple ground-glass-like high-density shadows on both lungs with blurred edges and interstitial changes on January 28. C) No significant difference from previous observation on January 31. D) Slightly absorbed shadow in the upper lobe on February 3. E) Some lesions in the lower lobe of both lungs were slightly absorbed on February 6. F) No significant difference from previous observation on February 8. Ground glass white patches are shown in each subgraph due to SARS-CoV-2 infection. With the progression of recovery, gradual reduction of white patches is observed.
